# Anti-SARS-CoV-2 antibody immunoreactivity profiles during COVID-19 recurrence

**DOI:** 10.1590/0037-8682-0106-2022

**Published:** 2022-10-24

**Authors:** Maria da Penha Gomes Gouvea, Isac Ribeiro Moulaz, Thayná Martins Gouveia, Karen Evelin Monlevade Lança, Bárbara Sthefany de Paula Lacerda, Beatriz Paoli Thompson, Jéssica Polese, Marina Deorce de Lima, Rodrigo Ribeiro-Rodrigues, José Geraldo Mill, Valéria Valim

**Affiliations:** 1 Universidade Federal do Espírito Santo, Hospital Universitário Cassiano Antonio Moraes, Vitória, ES, Brasil.; 2 Universidade Federal do Espírito Santo, Centro de Ciências da Saúde, Vitória, ES, Brasil.; 3 Universidade Federal do Espírito Santo, Centro de Ciências da Saúde, Departamento de Pneumologia, Vitória, ES, Brasil.; 4 Departamento de Saúde do Estado do Espírito Santo, Laboratório de Saúde Pública do Estado do Espírito Santo, Vitória, ES, Brasil.; 5 Universidade Federal do Espírito Santo, Centro de Ciências da Saúde, Centro de Doenças Infecciosas, Vitória, ES, Brasil.; 6 Universidade Federal do Espírito Santo, Centro de Ciências da Saúde, Departamento de Ciências Fisiológicas, Vitória, ES, Brasil.; 7 Universidade Federal do Espírito Santo, Hospital Universitário Cassiano Antonio Moraes, Divisão de Reumatologia, Vitória, ES, Brasil.

**Keywords:** COVID-19, SARS-CoV-2, Recurrence, Humoral Reactivity

## Abstract

**Background:**

This study aimed to evaluate IgG and IgM levels in COVID-19 recurrence.

**Methods:**

The serum antibody levels and clinical data from 73 healthcare workers with SARS-CoV-2 divided into seroconverted (n=51) and non-seroconverted (n=22) groups were assessed. The presence of specific anti-nucleocapsid (anti-N) IgM and IgG for SARS-CoV-2 was evaluated. IgG antibodies to the SARS-CoV-2 spike receptor-binding domain were used to confirm non-seroconversion in all negative anti-N.

**Results:**

Four recurrent cases displayed mild symptoms and were non-seroconverted until the recurrence of symptoms.

**Conclusions:**

Undetectable anti-nucleocapsid IgM and IgG levels may be correlated with symptomatic COVID-19 recurrence.

In December 2019, several cases of severe pneumonia caused by SARS-CoV-2 emerged in China and were associated with the onset of a severe respiratory disease termed coronavirus disease 2019 (COVID-19)^1^. After the first cases were reported, SARS-CoV-2 spread rapidly, reaching nearly every country and compelling the World Health Organization to declare a global pandemic^2^. Although other studies have suggested that the immune response to SARS-CoV-2 is similar to that observed during SARS-CoV and/or MERS-CoV, humoral and cell-mediated immune mechanisms leading to protection against SARS-CoV-2 infection are still not fully understood. It has been suggested that SARS-CoV-2 specific IgM and IgG antibody levels decline five months post-infection, leading to the hypothesis that protection against a second exposure to COVID-19 may be hindered^3^.

Although the number of confirmed re-infection cases is small, they have been reported in several countries^4^ ^,^ ^5^ ^,^ ^6^. Interestingly, patients with confirmed re-infection in the United States, Netherlands, and Ecuador displayed more severe symptoms during the second infection^7^ ^,^ ^8^ ^,^ ^9^. Conversely, in China and Belgium, symptoms associated with re-infection were milder^8^ ^,^ ^9^.

This study aimed to investigate antibody immunoreactivity and clinical characteristics during the first and second symptomatic COVID-19 infections confirmed by positive real-time polymerase chain reaction (RT-PCR).

We conducted a longitudinal follow-up study of healthcare workers at the University Hospital of the Federal University of Espírito Santo (HUCAM-UFES/EBSERH), Vitória, Brazil.

Participants with a first COVID-19 diagnosis based on a positive RT-PCR result for SARS-CoV-2 and clinical manifestation underwent serial blood collection for IgM and IgG antibody analysis on days 15, 30, 45, 60, and 90 (D15, D30, D45, D60, and D90), respectively, after the onset of symptoms of the first infection.

The clinical criteria for COVID-19 were defined according to WHO^2^, and the severity of the disease was categorized according to the patient’s clinical presentation as follows: 1) mild with only flu-like symptoms and 2) severe when presented with pneumonia or severe acute respiratory syndrome (SARS). A recurrent case was considered when individuals presented with symptoms followed by an asymptomatic period of at least 30 days and started to display COVID-19 symptoms again. These patients underwent a new RT-PCR test and antibody tests performed on days seven, 15, 30, and 60 after the appearance of symptoms of recurrence (D7, D15, D30, and D60, respectively).

Vials containing nasopharyngeal samples in the viral transport media were collected during the first and second symptomatic infections. All samples were processed in a biosafety cabinet class-II B2 and further processed for automated viral nucleic acid extraction using a Maelstrom 4800 Nucleic Acid Extraction System (Taiwan Advanced Nanotech Inc., Taiwan) TANBead Nucleic Acid Extraction Kit (Taiwan Advanced Nanotech Inc.), according to the manufacturer’s protocol.

The presence of specific IgM and IgG for SARS-CoV-2 was evaluated using a chemiluminescent microparticle immunoassay (CMIA), Abbott’s SARS-CoV-2 assay (Abbott Laboratories, IL, USA), using an ARCHITECT i1000SR immunoassay analyzer. Results > 1.4 for IgG or IgM (>1.0) were defined as seroconverted (Group A). Results under these limits were defined as non-seroconverted (Group B).

The titers of IgG antibodies to the SARS-CoV-2 spike receptor-binding domain (IgG-S) were determined using a chemiluminescent microparticle immunoassay (CMIA) according to the manufacturer’s instructions (SARS-CoV-2 IgG II Quant assay, Abbott Laboratories, Abbott Park, IL, USA). The assay was performed using an ARCHITECT i1000SR immunoassay analyzer. The results are expressed as arbitrary units/mL. Seropositivity was defined as a titer of ≥50 AU/mL.

Anti-nucleocapsid (anti-N) IgG and IgM antibodies were used for all participants at all time points. In those that were anti-N IgG or IgM negative, IgG antibodies to the SARS-CoV-2 spike receptor-binding domain (IgG-S) were tested.

A detailed standardized clinical questionnaire was administered on day 15 of the first and second symptomatic episodes to obtain demographic data, symptoms, severity, comorbidities, and treatment data. Symptoms were monitored during the follow-up. The asymptomatic period was defined as the period between symptomatic recovery from the first infection and the beginning of the second symptomatic infection.

Statistical analyses were performed using the IBM SPSS Statistics Program (Version 24). Data were presented as frequency, percentage, central tendency, and variability. The normal distribution of the sample was verified using the Shapiro-Wilk test, and a comparison between seroconverted and non-seroconverted groups was performed using Mann-Whitney’s non-parametric test and Chi-square test. The significance level was set at a p-value of < 0.05.

During June and July 2021, every employee who worked at HUCAM-UFES/EBSERH and tested positive for COVID-19 was invited to participate in this research. Seventy-three healthcare workers with positive SARS-CoV-2 RT-PCR test results were enrolled. The participants had a mean age of 39.8 (± 9.9) years, and 54 (74%) were female. Groups A (n=51) and B (n=22) were similar in terms of age, sex, and comorbidities (Table 1).

**TABLE 1:** Demographic profile of the seroconverted versus non-seroconverted groups.

	All participants (n=73)	Group A (n=51)	Group B (n=22)
**Age - mean (SD) years**	39.8 (9.9)	39.4 (9.6)	41.4 (10.7)
**Female, n(%)**	54 (74)	36 (70.6)	18 (81.8)
**Previous comorbidities, n (%)**			
Arterial hypertension	12 (17.6)	8 (16)	4 (22.2)
Diabetes mellitus II	3 (4.4)	2 (4)	1 (5.6)
Pre-obese	25 (36.7)	19 (38)	6 (33.3)
Obese	19 (27.9)	17 (34)	2 (11.1)
**Common symptoms, n (%)**			
Head, body, and/or throat ache	59 (80.8)	43 (84.3)	16 (72.7)
Hyposmia and/or anosmia	49 (67.1)	36 (70.6)	13 (59.1)
Fatigue	44 (60.3)	33 (64.7)	11 (50)
**Smoking cigarettes, n (%)**			
Smoker	2 (3.1)	2 (4.2)	0
Past smoker	9 (13.8)	6 (12.5)	3 (17.6)
**Alcohol consumption, n (%)**			
Occasionally	39 (53.4)	27 (52.9)	12 (54.5)
≥2 times/week	12 (16.4)	8 (15.7)	4 (18.2)
Sedentary lifestyle*, n (%)	32 (43.8)	23 (45.1)	9 (40.9)

Group A (seroconverted); group B (non-seroconverted). Seroconversion was considered the result of IgM or IgG anti-nucleocapsid of SARS-Cov-2 by chemiluminescence. *Defined as physical exercise less than three times a week. SD: Standard deviation.

After six months of follow-up, the RT-PCR results confirmed recurrence in four cases of COVID-19 (Table 2). The mean asymptomatic period between the two symptomatic episodes was 100 (±72) days. The symptomatic period ranged from three to 22 days and five to 19 days for the first and second infections, respectively. The average period between the two positive RT-PCR test results was 112 (± 65) days.

**TABLE 2:** Clinical characteristics of the COVID-19 patients at the first and second symptomatic events with time considerations.

Case	Age (years)	Comorbidities	Severity		Hospitalization (days)		Asymptomatic period (days)	Medications		Duration (days)		Symptoms		Interval between
			1st	2nd	1st	2nd		1st	2nd	1st	2nd	1st	2nd	RT-PCR tests (days)
1	34	Depression	Mild	Mild	No	No	39	Prednisone, Ibuprofen, Dipyrone	Dipyrone, Bilastine	22	13	Cough, Sore throat, Headache, Myalgia, Earache, Nasal Congestion, Fatigue	Sore throat, Nasal congestion, Earache, Fatigue, Anosmia, Ageusia, Chest pain	62
2	37	None	Mild	Severe	No	2	90	Ivermectin, Azithromycin	Ivermectin, Azithromycin	14	13	Cough, Fever	Myalgia, Dyspnea, Cough, Fever	106
3	50	None	Mild	Mild	No	No	192	Azithromycin	None	3	5	Cough	Anosmia, Cough	195
4	47	None	Mild	Mild	No	No	163	None	*	7	19	Anosmia, Hyposmia, Hypogeusia	Fatigue, Myalgia, Headache, Diarrhea, Cough, Anosmia, Ageusia, Nasal congestion, Chest pain	165

**RT-PCR:** real-time polymerase chain reaction.

All the patients presented with mild symptoms during the first infection. The most frequently reported symptoms were cough and headache in the first infection, whereas cough and anosmia predominated in the second infection. One patient (case 2) required hospitalization and oxygen supplementation during the second symptomatic episode. Demographic and clinical findings of the recurrent cases are presented in Table 1.

Antibody immunoreactivity data showed that IgM was not detected in 22 (30.1%) of the 73 subjects (non-seroconverted group). Conversely, an early humoral immune response after the first infection was reported in 51 (69.9%) of the 73 patients (seroconverted group). All recurrent cases (n=4) were reported in non-seroconverted subjects (p = 0.010). Although SARS-CoV-2 specific IgM, IgG, and IgG-S antibodies were undetectable during the first infection, IgM was detected in all four cases of re-infection, whereas IgG was detected in three of the four cases (Figure 1). All four individuals with recurrence were negative in both tests (anti-N IgG, IgM, and IgG-S).

**FIGURE 1:**
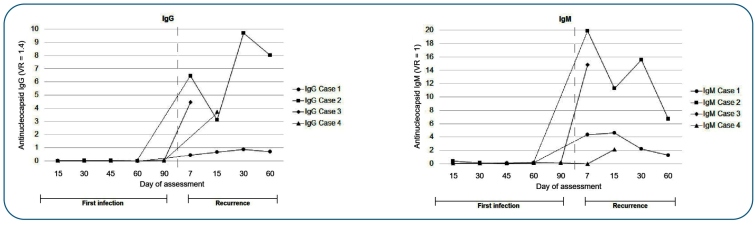
IgG and IgM immunoreactivity profiles after the first and second RT-PCR positive tests. IgG values ≥ 1.4 and IgM values ≥ 1 were defined as seroconverted.

In addition, Kaplan-Meier survival analysis indicated a significant difference between the seroconverted and non-seroconverted groups considering the time to the second RT-PCR positive test as the outcome, with all four recurrent cases among non-seroconverted individuals, while none were reported in the seroconverted group (p = 0.010). Antibody immunoreactivity data support the hypothesis that the lack of specific antibodies may increase the risk of SARS-CoV-2 re-infection (Mantel-Cox *chi*-square = 6.642) (Figure 2).

**FIGURE 2:**
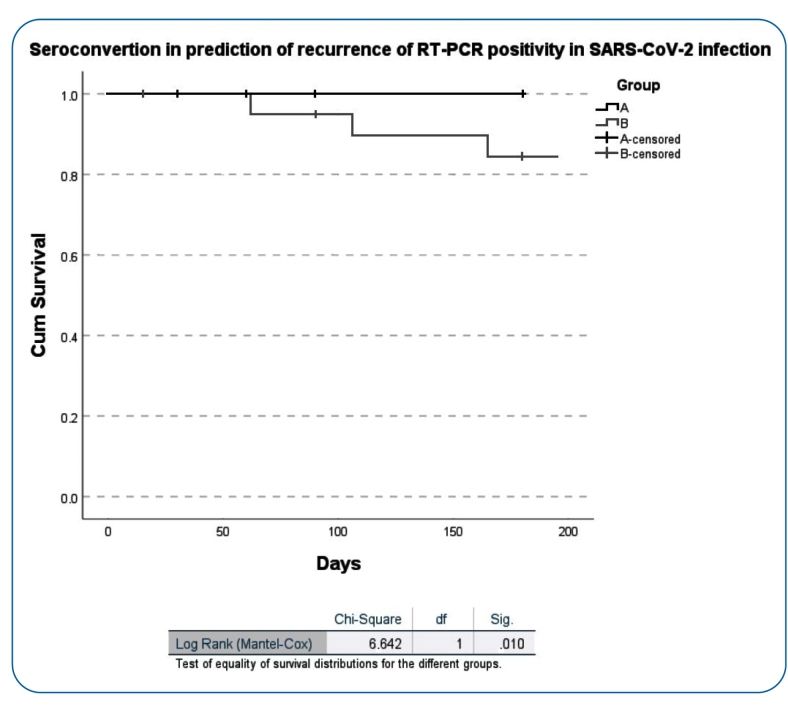
Kaplan-Meier curves - Comparison between seroconverted **(group A)** and non-seroconverted **(group B)**. The vertical ticks represent censored observations.

Although approximately 30% of the enrolled subjects did not present detectable levels of both IgM and IgG after the first symptomatic infection, the protective role of memory B cells against COVID-19 recurrence could not be excluded since these cells can be activated without the presence of detectable levels of immunoglobulins. Another possible explanation for undetectable levels of IgM and IgG could be the occurrence of ineffective antigen processing/presentation during the first symptomatic infection due to a low viral load, absence or reduced levels of circulating memory B cells, or even differential homing signaling of memory B cells to other lymphoid tissues. Moreover, antibody levels were correlated with age^10^ and disease severity^11^.

The prompt detection of both IgM and IgG antibodies may be due to rapid antibody production after antigen recognition by memory B cells elicited during the first infection. The duration of the response and effectiveness of memory B cells after SARS-CoV-2 infection remain unknown. Since humoral immunity usually fades out within a couple of months, determining the survival and effectiveness of memory B cells may further our knowledge about their role in protecting from re-infection cases.

Regarding COVID-19 severity, Jianghong An et al.^12^ reported that in a group of 262 patients followed for 14 days, 14.5% of young patients with mild SARS-CoV-2 infection were more likely to display a positive RT-PCR result after clinical discharge. Zheng et al.^13^ showed that 9.5% of 285 patients showed a positive SARS-CoV-2 test result at an average of seven days of follow-up. Re-infected patients generally displayed milder clinical symptoms, lower viral loads, shorter hospitalizations, and improved pulmonary conditions at readmission than during their first infection. However, opposite patterns during re-infection have also been reported^14^. In the present study, during recurrence, subjects presented with more severe symptoms than during the first infection. Although the correlation between the severity of SARS-CoV-2 symptoms during the first and second infection is still unclear, RT-PCR data (Ct values) showed that all four cases presented lower Ct values during COVID-19 recurrence, suggesting that re-infection was associated with a higher viral load. Therefore, the severity of symptoms observed during the second infection may be associated with a higher viral load. Considering that the number of recurrent cases in the present study was small, further investigations are required to confirm this hypothesis.

Although genomic sequencing is mandatory to confirm a re-infection case^7^, evidence such as a long asymptomatic period (39-192 days), a long interval between positive RT-PCR results (62-195 days), undetectable IgM and IgG antibodies after the first infection, and a prompt increase in IgM and IgG during the second symptomatic period are highly suggestive of re-infection. This was also observed in our study, with an average of 132 days between the two positive RT-PCR results and a 121-day asymptomatic period.

In the present study, samples for genomic sequencing were not available when the first symptomatic infection in the four cases was recorded. Nevertheless, sequencing data from samples collected in the state of Espírito Santo, which were available at the Brazilian Genomic Network, Brazilian Ministry of Health/FIOCRUZ^15^, showed that from June to August 2020, the period when the first COVID-19 infection was reported for cases #1-4, the B.1.1.33 variant was the predominant lineage; it was found in 100% of the sequenced SARS-CoV-2 samples. However, when cases of recurrent COVID-19 were confirmed in October 2020 (cases #1 and # 2), variants B.1.1.33, B.1.1.28, and P.2 represented 47%, 33%, and 20% of the sequenced samples, respectively. By January 2021, when recurrent cases #3 and #4 were identified, the diversity of circulating SARS-COV-2 augmented, with the identification of P.2, B.1.1.33, B.1.1.28, P.1 (Gamma), and B.1.1.7 (Alpha) variants, representing 56%, 20%, 14%, 8%, and 2% of the sequenced samples, respectively. Although the possibility of a second positive RT-PCR result due to viral reactivation or a low viral excretory mechanism could not be excluded, the time gap between the two RT-PCR tests and sequencing data describing the circulating variants in the state of Espírito Santo at the time provided additional evidence that a new infection by a different variant might have occurred.

Despite the limited number of enrolled patients (n = 73), the observed recurrence in four (18.2%) of the 22 subjects without detectable IgM/IgG after the first SARS-COV-2 infection suggests that natural infection may not induce similar protection in all exposed individuals. Further studies are needed to better understand antibody immunoreactivity after the second SARS-CoV-2 infection. Together, the data presented here emphasize the need to vaccinate the population, even with a previous history of natural infection.

In conclusion, patients with undetectable levels of IgG and IgM anti-nucleocapsid after a mild SARS-CoV-2 primary infection seem to be more prone to recurrence of severe symptomatic infection than those with detectable immunoglobulins.
